# Deep metabolome: Applications of deep learning in metabolomics

**DOI:** 10.1016/j.csbj.2020.09.033

**Published:** 2020-10-01

**Authors:** Yotsawat Pomyen, Kwanjeera Wanichthanarak, Patcha Poungsombat, Johannes Fahrmann, Dmitry Grapov, Sakda Khoomrung

**Affiliations:** a Translational Research Unit, Chulabhorn Research Institute, Bangkok, Thailand; b Metabolomics and Systems Biology, Department of Biochemistry, Faculty of Medicine Siriraj Hospital, Mahidol University, Bangkok 10700, Thailand; c Siriraj Metabolomics and Phenomics Center, Faculty of Medicine Siriraj Hospital, Mahidol University, Bangkok 10700, Thailand; d Center for Innovation in Chemistry (PERCH-CIC), Faculty of Science, Mahidol University, Rama 6 Road, Bangkok 10400, Thailand; e Department of Clinical Cancer Prevention, The University of Texas MD Anderson Cancer Center, 1515 Holcombe Boulevard, Houston, TX 77030, USA; f CDS- Creative Data Solutions LLC, https://creative-data.solutions, USA

**Keywords:** Metabolomics, NMR, Mass spectrometry, Artificial neural network, Deep learning, AI, Artificial Intelligence, ANN, Artificial Neural Network, AUC, Area Under the receiver-operating characteristic Curve, CFM-EI, Competitive Fragmentation Modeling-Electron Ionization, CNN, Convolutional Neural Network, CCS value, Collision Cross Section value, DL, Deep Learning, DNN, Deep Neural Network, ECFP, Extended Circular Fingerprint, ER, Estrogen Receptor, FID, Free Induction Decay, FP score, Fingerprint correlation score, FTIR, Fourier Transform Infrared, GC–MS, Gas Chromatography-Mass Spectrometry, HDLSS data, High Dimensional Low Sample Size data, IST, Iterative Soft Thresholding, istHMS, Implementation of IST at Harvard Medical School, LC-MS, Liquid Chromatography-Mass Spectrometry, LSTM, Long Short-Term Memory, ML, Machine Learning, MLP, Multi-layered Perceptron, MS, Mass Spectrometry, *m*/*z*, mass/charge ratio, NEIMS, Neural Electron-Ionization Mass Spectrometry, NMR, Nuclear Magnetic Resonance, NUS, Non-Uniformly Sampling, PARAFAC2, Parallel Factor Analysis 2, ReLU, Rectified Linear Unit, RF, Random Forest, RNN, Recurrent Neural Network, SMARTS, SMILES arbitrary target specification, SMILE, Sparse Multidimensional Iterative Lineshape-enhanced, SMILES, Simplified Molecular-Input Line-Entry System, SRA, Sequence Read Archive, VAE, Variational Autoencoder

## Abstract

In the past few years, deep learning has been successfully applied to various omics data. However, the applications of deep learning in metabolomics are still relatively low compared to others omics. Currently, data pre-processing using convolutional neural network architecture appears to benefit the most from deep learning. Compound/structure identification and quantification using artificial neural network/deep learning performed relatively better than traditional machine learning techniques, whereas only marginally better results are observed in biological interpretations. Before deep learning can be effectively applied to metabolomics, several challenges should be addressed, including metabolome-specific deep learning architectures, dimensionality problems, and model evaluation regimes.

## Highlights

• The applications of deep learning has recently emerged in metabolomics research.
• Deep learning has been most widely applied in data pre-processing step.
• Convolutional neural networks are the most commonly used model architecture.
• Development of deep learning for metabolomics is not as mature as that for genomics.

## Graphical abstract

**Figure:**
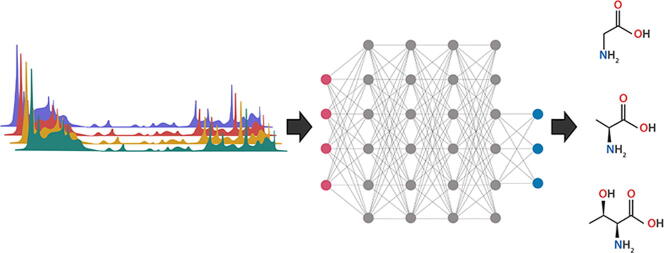

## 1 Introduction

Machine learning (ML) or the concept of ‘training’ computational methods which can improve given more ‘experience’ or data has been a revolutionizing force in many disciplines, including metabolomics, for the last 15 years. In particular, deep learning (DL) [1], an ML method based on artificial neural networks (ANN) has been increasingly applied to problems in metabolomics, which are very difficult or infeasible for conventional algorithms to solve. For example, in nuclear magnetic resonance (NMR) and mass spectroscopy (MS) based metabolomics, a variety of ML algorithms have been developed for data pre-processing, peak identification, peak integration, compound identification/quantification, data analysis, and data integration [2], [3], [4], [5], [6]. ANNs are part of a broad family of ML algorithms that seek to learn rules/conditions from data examples, and in some cases can be ‘automatically’ improved through the sheer amount of data available to the model training process[7]. Ease of use and accessibility of ANN and DL methods are increasing for the metabolomics community due to development of neural network frameworks (such as TensorFlow [8], [9], Keras [10], PyTorch [11]), simplified interfaces to the frameworks through high-level programing languages (such as Python [12], R [13], MATLAB [14]), and reduction in model computational time through optimization using graphics processing units (GPUs), which can effectively parallelize complex tasks (e.g. matrix multiplication) and are readily available through stand-alone graphics cards in workstation-class machine or cloud computing services (Amazon Web Service [15], Google Cloud Platform [16], Microsoft Azure [17]).

ML is part of the broader domain of artificial intelligence (AI). In traditional programing, predefined sets of rules (i.e. algorithms) are applied to the data to produce desired output. However, in ML, a portion of data and examples of desired output are used to train a model (i.e. to derive rules from the data), which can then be applied to make predictions on other data. Unlike traditional ML methods that focus on feature engineering (i.e. transforming raw data into features that are relevant for machine learning models [18]), ANN and DL emphasize on tuning model hyperparameters. The ability of these methods to both encode and model the data removes a large bottleneck and source of potential bias for traditional ML algorithms. ANNs have simple structures consisting of three layers of neurons: input, hidden, and output layers. Each input neuron is connected to every hidden layer neuron by an edge which defines a weight and a bias. Inspired by how neurons function in the brain, each artificial neuron will emit (fire) a response depending on the activation function. For example, if a signal from an input layer neuron, combined with specific weight and bias, is higher than a certain threshold set by an activation function, then the neuron will send out a signal to the output layer [19]. ANN and DL models differ based on their architectures (i.e. number of layers and their connections) and structures with less than two hidden layers are called shallow ANNs, while more complicated architectures are found in the larger class of Deep Neural Network (DNN) which can be more expressive and efficient than their simpler ANN variants [20]. For reviews of introductory ANN and DL methodology, which is outside the scope of this review, we refer readers to other articles containing historical and methodological perspectives [21], [22].

## 2 Landscape of deep learning in metabolomics

**Fig. 1:**
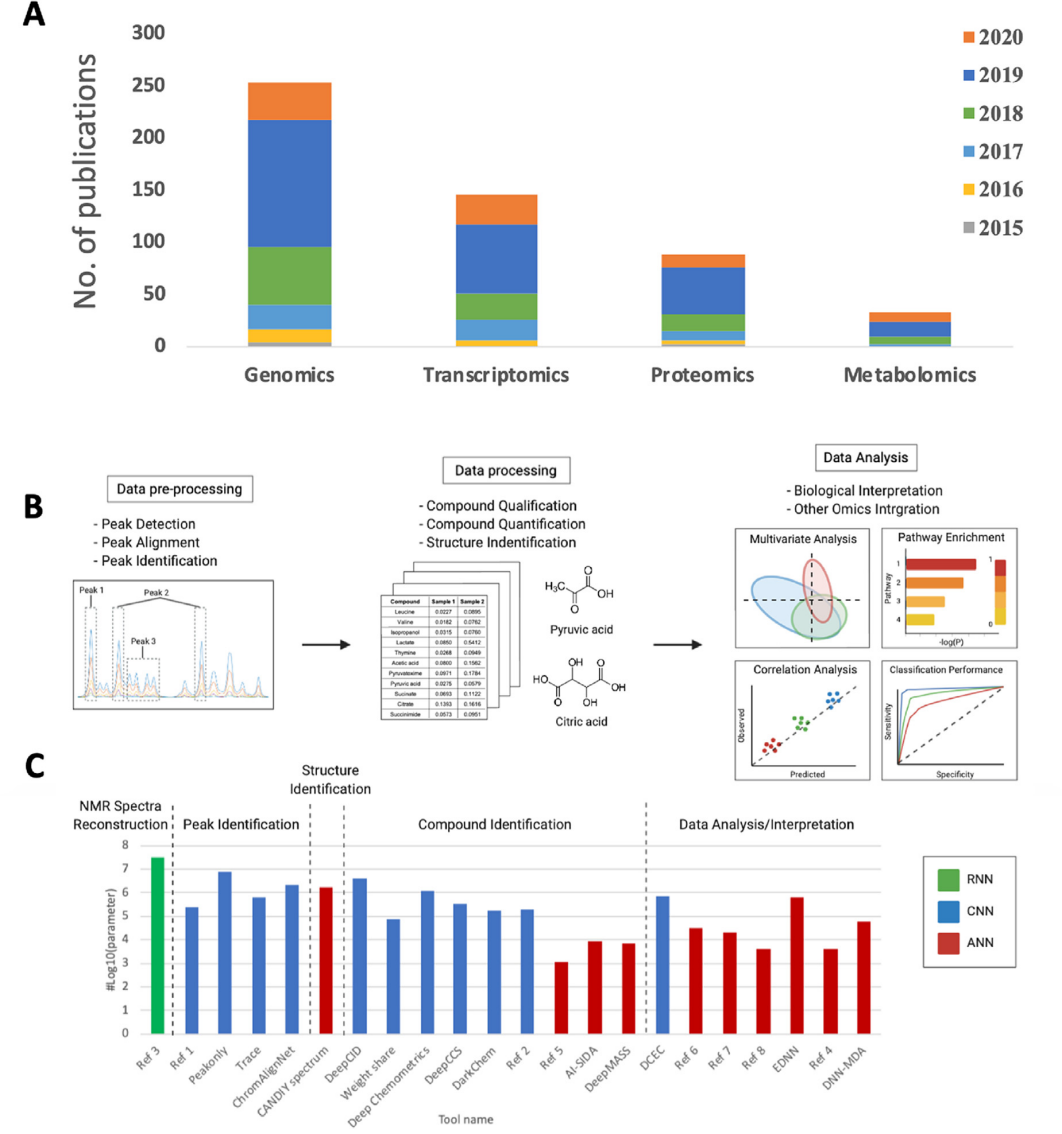
A) Number of publications with the keyword “deep learning” extracted from PUBMED database from 2015 to April 2020 in the genomics, transcriptomics, proteomics, and metabolomics. B) Three categories of metabolomics application that have applied deep learning. C) Barplot of the number of parameters based on different neural network architectures and applications. RNN, recurrent neural network; CNN, convolutional neural network; ANN, shallow artificial neural network.

The number of publications from PubMed search results with DL as one of the keywords (as of May 2020) in genomics, transcriptomics, proteomics, and metabolomics are shown in Fig. 1A. Note that the number of DL-associated publications in metabolomics are significantly lower than all other omics. The following review focuses on applications of ‘Artificial Neural Networks’, ‘Deep Neural Networks’ or ‘Deep Learning’ on MS or NMR based measurement of metabolites and small molecules and is divided into the three following domains: I) peak alignment and identification; II) structural/compound identification and quantification; and III) data analysis, interpretation, and integration with other omics (Fig. 1B). Numbers of hyperparameters of DL models used in these studies were calculated and shown in log-10 scale in Fig. 1C based on the number of neurons, number of neuron layers, and type of model architecture described in the studies. Notice that the number of parameters in data pre-processing applications are generally higher than other applications, and studies that used CNN architecture tend to have higher parameters than shallow ANN architecture. The most popular DL framework reviewed is Keras [10], which can use TensorFlow [8], Theano [23], or PlaidML [24] as backends to generate and run models. Python [25] were the most popular programming language interfaces for DL frameworks, followed by R [26] and MATLAB [27]. Close to half of the studies reviewed utilised GPUs (typically one GPU on a workstation-class machine). In addition to Keras, other DL frameworks used in metabolomic applications included: H2O.ai [28], MXNet [29], and MATLAB’s Deep Learning Toolbox [30]. All reviewed frameworks, backends, programing languages, and types of processing units are summarised in Table 1. The data source (i.e. biological samples), data types, URLs for raw data access and code depositories of articles reviewed in this manuscript are summarised in Supplementary Table 1.

**Table 1:** All reviewed frameworks, backends, programing languages, and types of processing units are summarised.

		Peak Alignment/Identification	Compound and Structure Identification/Quantification	Data Analysis/Omics Integration	Total
Framework**	Keras	2	5	3	10
	MXNet	0	0	1	1
	H2O*	0	0	2	2
	FANN/RPROP	0	1	1	2
	DLT	1	2	0	3
Backend	TensorFlow	3	7	2	12
	Theano	0	1	1	2
	PyTorch	1	0	0	1
	MXNet	0	0	3	3
	Others	1	3	3	7
Programming Language	Python	4	8	3	15
	R	0	0	2	2
	MATLAB	1	2	0	3
	C	0	1	1	2

DLT = Deep Learning Toolbox in MATLAB, note that this also includes old implementation Neural Network Toolbox

Others = MATLAB toolboxes, RPROP

* Since 2018, H2O no longer uses MXNet or TensorFlow as backend. As these studies were conducted prior to 2018, we assumed (according to the source code) that the framework still employed default backend, which is Apache MXNet)

** Some studies employed TensorFlow directly as their framework in Python. Therefore, the number of studies in framework rows are not matched with number of Backend rows.

Convolutional neural networks (CNN) were the most often utilised DL model architecture across all metabolomics data pipeline steps. These models are often used in image processing due to their shift invariant characteristics and their application to metabolomic data varied across model complexities (e.g. numbers of neurons, hidden layers, filters, different types of optimizers, activation functions and loss functions). While many of the reviewed studies employed multiple types of neural networks in their work, including for different steps or performance comparisons, the non-linear rectified linear unit (ReLU) [31] was the most widely used activation function. This is not surprising because ReLU is generally the most widely used activation function particularly for CNNs [32] and may offer some advantages for dealing with the sparse nature of metabolomics data. All of the reviewed peak alignment applications included CNNs as the core architecture or part of the workflow. DL model architectures for other workflow steps included a mix of shallow ANN and other variants of DNN such as autoencoders and CNNs (Fig. 1C).

## 3 DL in NMR spectra processing and interpretation

Nuclear magnetic resonance (NMR) spectroscopy is a prevalent technique for metabolomics analysis owning to its advantages i.e. non-destructive, fast, accurate, able to detect most of organic compounds, and highly reproducible when compared with MS [33], [34]. The common step in NMR data handling begins with data pre-processing to transform the free induction decay (FID) to matrix of chemical shift and its intensity. The baseline correction, normalization, and alignment are subsequently performed before metabolite quantification and statistical analysis i.e. multivariate or univariate analysis [35]. NMR is widely used in metabolomics for both qualitative and quantitative analyses [36]. One-dimensional (1D) ^1^H, and ^13^C NMR are the two most commonly used methods for measuring primary metabolites. Depending on structural complexity and surrounding environment of the compounds being measured (e.g. natural products), two-dimensional (2D) NMR is often considered as the technique of choice [37], [38]. Although there are multiple steps of data processing and analysis, most applications of DL in metabolomics were for signal processing. This may be in part due to the large data requirements for DL for which simulation or synthetic data creation have been proposed [39]. Hansen (2019) proposed to use DNN to reconstruct non-uniformly sampling (NUS) NMR spectra. To improve accuracy in spectrum intensity, the author built DNN that was inspired by long short-term memory (LSTM) networks with a series of 8x10^6^ synthetic one-dimensional FIDs (free induction decays) to reconstruct the DNN model. The model was later validated and compared against sparse multidimensional iterative lineshape-enhanced (SMILE), hmsIST algorithms by using the experimental ^15^N-^1^H HSQC spectrum. The DL-based approach showed equally good or slightly better NMR spectra reconstruction results compared with current state of the field methods [40], [41] proposed to use a CNN to reconstruct fast and high-quality NMR spectra of small and large (metabolites) and small proteins from fully simulated NMR data [41]. The model performance was validated with an input of 2D ^1^H-^15^N HSQC spectrum with 25% NUS data quality against the fully sampled 2D and 3D spectra and obtained a correlation of peak intensities of 0.99. This model also displayed correlation coefficient greater than 0.98 to 2D spectra [41] even in low-density regions.

## 4 DL in MS spectra processing and interpretation

Mass spectrometry (MS)-based metabolomics measures the mass-to-charge (*m*/*z*) ratio and corresponding intensities of metabolite species in a sample. A raw MS data file of an individual sample contains a set of chromatograms recorded in sequence. Literally, each chromatogram, consisting of mass spectra or fingerprint of the detected metabolite represents the abundance of an ionized molecule [42]. Raw data files are subjected to a series of data processing steps and information extraction into an expression matrix (containing retention times, accurate mass spectrum and intensity values) of the measured metabolites for subsequent analyses [43].

Raw MS-based data processing is a critical step that can affect quality of downstream analyses and interpretation of metabolomics data. General MS data pre-processing steps include noise filtering, peak detection, peak alignment and normalization [44], [45], [46]. Data filtering is to remove or reduce analytical noise or baseline. Peak detection distinguishes real signals of measured molecules from noise. Peak (feature) alignment is an effort to correct retention time shift across different samples, and data normalization removes systematic variations between samples. Numerous free and commercial software are available for MS-based data processing such as MZmine [47], XCMS [45], metaMS [48], and metAlign [49], to name a few. However, key challenges, such as false positive signals, co-eluting compounds and non-linear retention time shift, still need to be addressed [50], [51], [52]. With the complexity of MS data, DL approaches have been proposed to solve this key data pre-processing step and major bottleneck of MS-based metabolomics pipelines. A study from Risum et al. [53] used CNN to classify different elution profiles from raw GC–MS chromatographic data. These profiles were initially modelled by PARAllel FACtor analysis2 (PARAFAC2) [50], [54] and subsequently delineated into chemical peaks (metabolite), baselines and other non-related peak areas by the CNN model, which resolved which peak component were most suitable for selection or integration. Similarly, Melnikov et al. proposed ‘peakonly’ algorithm [51] for both peak detection and integration that used a CNN model to classify raw LC-MS data into regions of noise, chemical peaks, and uncertain peaks, which was then used to determine peak boundaries for integration. Automated and high accuracy peak classifiers would greatly improve efficiency in these critical steps, which often heavily rely on domain experts.

Peak alignment is commonly performed to address retention time shift in MS methods employing chromatographic separations. Li and Wang et al. proposed ChromAlignNet [55], which uses LSTM network (a variant of recurrent neural network (RNN)) for peak alignment of GC–MS data. They showed that the algorithm performed well for the alignment of complex GC–MS data without the need for additional parameter selection and reference chromatograms. Discrimination of true chromatographic peaks from noise is particularly challenging. DL based peak filtering approaches seek to overcome the limitations of traditional methods for handling low signal to noise, diverse and irregular peak shapes and poor baseline resolution. For example, Kantz et al [56] used a CNN model to detect true spectral peaks vs. artifacts using stacked peak images of LC-MS chromatographic features as input data. This approach was shown to eliminate up to 90% of all false noise peaks. The versatility of DL models for encoding and modeling diverse forms of data have increased their adoption among the dominant metabolomics methods including LC-MS, GC–MS, and NMR.

Small molecule structure identification remains one of the biggest challenges in metabolomics (particularly for MS-based methods). Typically, retention time, accurate mass and mass spectra acquired from various analytical platforms are searched against reference databases [57], [58], [59] such as HMDB [60], METLIN [61] and MassBank [62] to name a few. Similarities between unknow and reference compounds’ data are typically estimated based on correlation [63], weighted cosine similarity [64] and Euclidean distance[65] which are used to rank the matching candidate hits [66]. This approach is limited by availability of known compounds and their spectral coverage in the reference databases [67]. Recently, Fan et. al. [68] used a CNN for identification of components in raw Raman spectra of mixtures without the need for any prior spectral processing which can otherwise introduce variability and errors. In another DL-based structure identification example, Fine et. al. [69] applied an autoencoder to calculate a lower-dimensional encoding of Fourier Transform Infrared (FTIR) and MS data together with multi-layered perceptron (MLP) to predict functional groups. A similar approach was used by Lim et. al. [68] to elucidate candidate structures using a CNN classifier to predict the presence/absence of substructures based on compounds’ mass spectra and chemical formula. DeepMass, presented by Ji et. al. [59], addresses the limitation of availability of spectra in the reference databases by increasing the chance to identify unknown compounds by augmenting the search results based on structural similarity to related known metabolites. The developed method leverages structural similarity between biochemical reactant and product pairs’ substructures and their resultant mass spectra [70]. The authors used KEGG substrate-product pair information to determine structural similarity scores between pairs of unknown-known metabolites (MASS score) from their MS/MS spectra. The authors then calculated fingerprint correlation score [71] (FP score), another structure similarity score for unknown-known structure matching, to compute the final list of putative compounds. Other studies, such as those from Allen et. al. [72] and Wei et. al. [73] attempted to increase spectral library coverage by predicting MS spectra for small molecules. Competitive Fragmentation Modeling-Electron Ionization (CFM-EI) [72] from Allen et al. used a probabilistic Markov model together with ANN to predict the tendency of bond breaking in a molecule and fragmentation likelihoods resulting in spectral peaks. Meanwhile, Wei et. al. [73] employed a MLP to predict MS spectra for small molecules. The input data for the bidirectional prediction model, Neural Electron − Ionization Mass Spectrometry (NEIMS) model, is the mapped additive Extended Circular Fingerprints (ECFPs), which capture local structures in the molecule [74] and the model output is a vector of intensity at all *m*/*z* bins.

ML algorithms including DNN models have also been used to predict collision cross section (CCS) value [57], [67], [75], [76], a chemical property of ion separation that can be directly obtained from ion mobility-MS (IM-MS)[70]. The CCS is exploited to narrow down the search space for unknown compound identification [77]. Given that CCS information is still limited, Plante et. al. [67] proposed CNN-based model (DeepCCS) for predicting the CCS value of a compound given the simplified molecular-input line-entry system (SMILES) representation and the ion type. Colby et. al. [76] generated a model, DarkChem, built from a variational autoencoder (VAE) architecture for predicting *m*/*z* and CCS values of specified molecular structures, as well as computing possible structures from given chemical properties. The predicted CCS values can be used in addition to spectral database matching to increase confidence while performing compound matching [57], [58].

## 5 Biological data interpretation & integration with other 'omics

Post data pre-processing, metabolomics data can be represented in a tabular format (e.g. samples as rows and measured metabolites as columns) making it amenable for a variety of downstream data analyses or ML tasks. For example, data normalization, outlier detection, missing values imputation and feature selection are a few common analyses often conducted prior to ML modeling [78]. Analyses specialized for metabolomics data often utilise information about the measured species’ structural and or biochemical properties to improve the biological and systems biology interpretation of the results (e.g. pathway enrichment, structural similarity or biochemical precursor to product networks).

Metabolomics datasets are often ‘wide’ (i.e. samples ≪ measurements) which poses significant challenge for ML applications which require abundant samples or representations for training and validation. For example, ML model validation often involves splitting the data representations into a training (used to build model) and test (used to validate model performance) sets. Other challenges include many highly correlated variables which can be the outcome of linked biochemical processes, but pose challenges for predictive modeling (multicollinearity) which can make identification of important biomarkers (feature selection) less robust. Metabolomics specialized ML approaches include methods incorporating dimensional reduction (e.g. PCA) with classification or regression models (e.g. PLS-DA, OPLS, etc). However, these non-DL methods generally cannot model non-linear relationships and are highly sensitive to noise and outliers. Given enough representations, the expressivity of DL architectures can be used to build internal representations of the data which may lead to superior predictive performance compared to non-DL approaches.

Analysis requirements may encompass both supervised (regression and classification), semi-supervised (partially labeled data) and unsupervised (e.g. clustering) tasks. Supervised and semi-supervised methods are used to predict known values (labels) such as sample groups (classification) or continuous values (regression) given samples’ metabolic profiles. Unsupervised algorithms do not required labels and are instead used as unbiased methods to group (cluster) and explore the data. Herein, we present a few applications comparing binary (two-class) and multi-class classification, and regression using several types of DL models.

DL has not been shown to be superior to other ML methods for predictive modeling tasks in metabolomics. For example, two DL and six ML algorithms for binary classification across ten clinical metabolomics datasets were compared [79]. While DL-based predictions on test data yielded good to excellent classifier performances, no single DL or ML algorithm could be identified as superior [79]. In another study, Bahado-Singh and colleagues evaluated the application of DL techniques to amniotic fluid metabolomics and proteomics alone and in combination with sonographic, clinical and demographic information to predict obstetric outcomes in asymptomatic pregnant women with short cervical lengths [80]. The authors further compared classifier performance derived from DL to that of six commonly used ML techniques. Higher area under the receiver-operating characteristic curve (AUC) point estimates were consistently achieved with DL in comparison with that of the other ML methods [80]. A third study examined the accuracy of feed-forward networks, a type of DL framework, as well as six widely used ML models to predict ER status based on a publicly available metabolomics data set [81]. The DL framework yielded the highest AUC point estimate for classifying ER+/ER- subjects based on metabolomics data compared to that of the other six ML algorithms. Importantly, biological interpretation of the first hidden layer identified by the DL framework revealed enrichment of eight cancer-relevant metabolic pathways that were not identified through the conventional ML algorithms [81]. Although the authors caution that the classifier performance of the DL method was very sensitive to sample size and discretion should be used when applying DL methods to small sample sets [81]. Wang and colleagues utilised SMARTS-encoded metabolic reaction rules to extract molecular fingerprints and, using these fingerprints, employed DL algorithms to interrogate drug metabolism and predict those biochemical reactions that are most likely to occur [82]. Performance of the DL algorithm was additionally compared against the rule-based method SyGMa [83]. In the test set, the DL algorithm achieved an accuracy of 78% for the top-10 common metabolic reactions, which was substantially improved relative to the SyGMa method (accuracy of 70%).

Only one study used DL in multi-class classification for classifying three types of heart disease, adenocarcinoma status, and three polymorphisms of NOS1AP genes from untargeted GS-/LC-MS data [84]. In this study, DL was no better than convention ML methods [84]. For linear regression, one study used an ensemble DNN approach to predict fish size from metabolites measured by NMR [85]. Like the aforementioned study, using DL-based regression to model the relationship between fish sizes and their metabolic profiles yielded a model with comparable performance to that of a traditional ML, Random Forest (RF) model, [85]. These authors note, however, that disparity in DL model performance could be due to the simplicity of the DL architectures used to analyze heterogeneous and complex data or due to limitations in sample numbers which were required for the neural network to separate the signal from the noise.

## 6 Future perspectives and beyond

### 6.1 Data integration applications are still lacking

Despite several publications on multi-omics data integration using deep learning [86], [87], there are only two studies which we could identify that directly combined metabolomics with other omics data. The first study aggregated temporal proteomics and metabolomics data of cardiovascular mouse models, and then used DL-based clustering methods to identify biologically relevant clusters of metabolites that linked to the conditions [88]. The second study integrated a large compendium of multi-omics data from *E. coli* to predict its cellular state. While a myriad of DL methods have been proposed as candidates for multi-omics data integration [89], few metabolomics data sets have been successfully incorporated into the models. The reasons for this deficiency may include a lack of data availability, especially human-centric data. For comparison, Sequence Read Archive (SRA) database, a repository for next-generation sequencing data, has ∼ 1,000 human-related studies [90], whereas Metabolomics Workbench database has 68 human-related projects [91]. Ultimately, this might be due to different cost structures between genomics [92] and metabolomics [93], [94], as well as lack of interdisciplinary research opportunities among deep learning practitioners, metabolomics experts, and other omics scientists [93].

### 6.2 Dealing with the curse of dimensionality

**Fig. 2:**
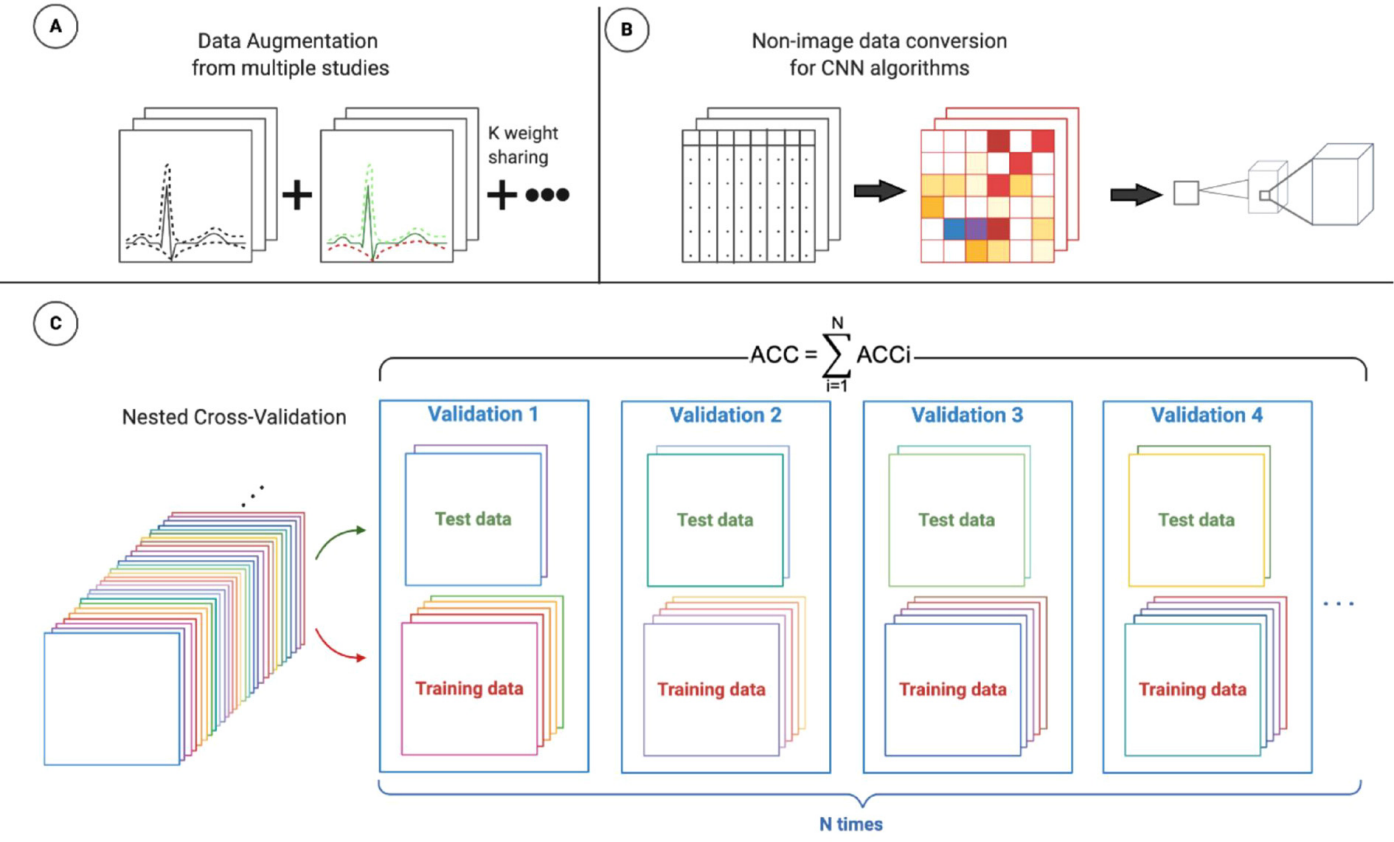
A) Combination of data augmentation and weight sharing from different studies can alleviate dimensionality problem in metabolomics. B) Biological data interpretation could benefit from non-image data conversion to leverage the power of CNN architecture. C) Model evaluation should employ nested cross-validation instead of conventional k-fold cross validation.

Low numbers of samples compared to many measured features leads to the curse of dimensionality, where predictive models can be overfitted and are not able to generalise to other data sets [22], [95]. A few methods that can overcome this limitation include data augmentation and weight sharing (Fig. 2A). For example, a study employed data augmentation, where original near infrared spectra were modified by adding random variations in offset, slope, and multiplication to improve robustness of the neural network models [96]. Another study introduced a concept called weight sharing, where two or more data sets are subjected to the same CNN architecture without resizing dimensions of one data set to fit the other, and the weights are shared during the training process [97]. Other methods have also been specifically designed for high dimensional low sample size data (HDLSS). For example, the Deep Neural Pursuit model designed specifically for genomics data, can be used for feature selection from a subset of samples combined with multiple dropouts technique [98] to reduce overfitting which may also benefit modeling of metabolomics data.

### 6.3 Specialized models for metabolomics data are needed

Compared to genomics, DL applications in metabolomics lack custom features that take advantage of specific properties of metabolomics data. Several comprehensive reviews of DL in genomics and proteomics showed well-defined problem statements and methods, which utilised unique approaches purposefully built for genomics applications [86], [87]. Such examples in genomics are CNN models for DNA/RNA binding motif prediction [99], [100] and functional non-coding sequence variant prediction [101]. These methods employed a strategy that converts DNA/RNA sequences into one-hot encoded representations that are suitable for CNN architecture. Protein contact map prediction also benefited from the strategy previously described, where protein sequence and predicted structure profiles (α-helix, β-strand, and loop region) are transformed by one-dimensional (1D) CNN to two-dimensional (2D) matrix, and then parsed to 2D CNN. DL analysis in metabolomics, especially biological interpretation, could benefit from a similar strategy, by converting non-image data to image-like data which is suitable for CNN [102] (Fig. 2B).

### 6.4 Re-evaluating model validation

Model accuracy and generalisability are often major priorities for ML applications. However, even with data augmentation and multiple data sets integration, compared to other domains, metabolomics data still lacks the sheer number of samples used in standard machine learning applications [103]. Even some of the standard techniques used for model validation such as *k*-fold cross-validation [43], [104] may not be applicable for HDLSS metabolomics data. For example, some studies cautioned that random data splitting techniques like *k*-fold cross-validation might yield overfit and unstable models [79] from HDLSS data. To address this issue, researchers have developed an alternative technique called nested cross-validation [105] (Fig. 2C). A recent simulation study showed that models trained with nested cross-validation yielded unbiased performance even with small sample sizes [106].

In conclusion, DL is starting to make a significant impact on metabolomics data processing and analysis pipelines. The application of DL in both NMR- and MS-based metabolomics is expected to grow rapidly as the metabolomics community begins to implement and develop novel DL architectures specific to metabolomics data applications.

## 7 Key points

• While machine learning has been used in metabolomics for decades, the application of artificial neural networks and particularly deep learning has only recently emerged.
• Deep learning has been most widely applied in data pre-processing and convolutional neural networks are the most commonly used model architecture.
• Development of deep learning applications specifically for metabolomics is not as mature as that for other omics domains such as genomics.
